# Miscellany

**Published:** 1905-06

**Authors:** 


### Miscellany.

An Adjustable Indicator, for accurately measuring small quantities of the more volatile anæsthetics, invented by Mr. Vernon Knowles, consists of a flat metallic bar provided with clips engaging the bottom and the neck of a glass bottle for the purpose of securely holding it in a vertical position against the side of the bottle. Attached to, and moving on this bar, are two small curved arms or wings, controlled by a set-screw. One arm is graduated by means of steps for cubic centimetres, 1 to 5, and the other for the divisions of one dram.

Before administration, one of the wings should be adjusted by means of the set-screw, so that the upper edge of the same is on a level with the fluid. If this precaution be taken, the danger incurred by an "overdose" is entirely eliminated, because, as the anæsthesia proceeds, the administrator has only to glance at the indicator, when he can tell exactly the dose that has been given. Having the graduation marked by steps cut out of the metal, instead of marks made upon its surface, is an excellent idea, they are so much more readily seen.

---

New Grip Gags, to keep the mouth open when administering anæsthetics, and which may be used while the face-piece is on, are advertised by Taylor's Dental Depot, 17 Poland Street, London, W. These are very much like a pair of pliers, held open by a ratchet, and provided with pads to rest against the teeth.

